# Selection of a Novel Anti-Nicotine Vaccine: Influence of Antigen Design on Antibody Function in Mice

**DOI:** 10.1371/journal.pone.0076557

**Published:** 2013-10-01

**Authors:** David C. Pryde, Lyn H. Jones, David P. Gervais, David R. Stead, David C. Blakemore, Matthew D. Selby, Alan D. Brown, Jotham W. Coe, Matthew Badland, David M. Beal, Rebecca Glen, Yvonne Wharton, Gavin J. Miller, Phil White, Ningli Zhang, Michelle Benoit, Karen Robertson, James R. Merson, Heather L. Davis, Michael J. McCluskie

**Affiliations:** 1 Pfizer Worldwide Medicinal Chemistry, Cambridge, United Kingdom; 2 Pfizer Worldwide Medicinal Chemistry, BioTherapeutics Chemistry, Cambridge, Massachusetts, United States of America; 3 Pfizer Vaccine Research, La Jolla, California, United States of America; 4 Pfizer Worldwide Medicinal Chemistry, Groton, Connecticut, United States of America; 5 Pfizer Vaccine Research, Ottawa, Ontario, Canada; 6 Peakdale Molecular Ltd, Chapel-en-le-Frith, United Kingdom; 7 Pfizer Chemical Research and Development, Sandwich, United Kingdom; The Scripps Research Institute, United States of America

## Abstract

Anti-nicotine vaccines may aid smoking cessation via the induction of anti-nicotine antibodies (Ab) which reduce nicotine entering the brain, and hence the associated reward. Ab function depends on both the quantity (titer) and the quality (affinity) of the Ab. Anti-nicotine vaccines tested previously in clinical studies had poor efficacy despite high Ab titer, and this may be due to inadequate function if Ab of low affinity were induced. In this study, we designed and synthesized a series of novel nicotine-like haptens which were all linked to diphtheria toxoid (DT) as carrier, but which differed in the site of attachment of linker to nicotine, the nature of linker used, and the handle used to attach the hapten to DT. The resulting hapten conjugates were evaluated in a mouse model, using CpG (a TLR9 agonist) and aluminum hydroxide (Al(OH)_3_) as adjuvants, whereby Ab titers, affinity and function were evaluated using a radiolabeled nicotine challenge model. A series of additional linkers varying in length, rigidity and polarity were used with a single hapten to generate additional DT-conjugates, which were also tested in mice. Conjugates made with different haptens resulted in various titers of anti-nicotine Ab. Several haptens gave similarly high Ab titers, but among these, Ab affinity and hence function varied considerably. Linker also influenced Ab titer, affinity and function. These results demonstrate that immune responses induced in mice by nicotine-conjugate antigens are greatly influenced by hapten design including site of attachment of linker to nicotine, the nature of linker used, and the handle used to attach the hapten to DT. While both Ab titer and affinity contributed to function, affinity was more sensitive to antigen differences.

## Introduction

Tobacco use is responsible for approximately six million deaths annually and poses a substantial burden on public health worldwide [1]. While the vast majority of adult smokers wish to quit, approximately 80% of those who attempt to quit on their own will relapse within the first month of abstinence and less than 5% will remain abstinent at 6 months [2]. Pharmacological treatments currently used for smoking cessation are most often nicotine replacement therapies (e.g., gums, patches) or prescription drugs that act within the central nervous systems to reduce nicotine reward and/or symptoms of withdrawal. These treatments are beneficial for promoting short-term abstinence but are only modestly effective over the long-term, with fewer than one-quarter of treated subjects remaining abstinent at the end of one year [3,4].

Vaccines targeting nicotine are being developed as an alternative approach to treat nicotine dependence. For these vaccines, the putative mechanism of action is that they will induce anti-nicotine antibodies that bind nicotine in the periphery, thus reducing the amount of nicotine entering the brain, which should in turn reduce reward and help break the addiction cycle [5]. Clinical trials have been conducted with several different anti-nicotine vaccines that comprise nicotine-like haptens conjugated to different carriers, and an adjuvant that is most often an aluminum salt. Two vaccines have undergone phase 2 clinical testing as monotherapies, namely NicQβ (Cytos Biotechnology), which uses a virus-like particle as carrier [6] and NicVax (Nabi Biopharmaceuticals) which uses a bacterial exoprotein as carrier [7]. In both studies, there was a lack of efficacy in the intent to treat (ITT) population, however subgroup analyses showed enhanced long-term abstinence rate compared to placebo in smokers with the highest antibody levels [6,7]. This indicated that better overall vaccine efficacy might be achievable if high antibody levels could be generated in a greater proportion of subjects.

In previous studies, we evaluated a model anti-nicotine vaccine comprised of trans-3̕-aminomethylnicotine (3̕AmNic) conjugated to diphtheria toxoid (DT) and showed that using two adjuvants, namely aluminum hydroxide and CpG oligodeoxynucleotides (CpG, an agonist for Toll-like receptor 9), induced significantly higher anti-nicotine antibody levels in mice and non-human primates than when aluminum hydroxide was used as sole adjuvant [8]. When Ab function was evaluated in mice by intravenous (IV) administration of radiolabeled nicotine there was only a modest (30%) reduction of nicotine in the brain compared to non-vaccinated controls, even with the superior adjuvant formulation that gave approximately 10-times higher antibody levels. Function was assessed in non-human primates by determination of the nicotine binding capacity and when nicotine was added at a concentration that can be found in arterial blood of a chronic smoker (100 ng/mL), only 30% was bound, indicating that 70% would be available to enter the brain. A 30% reduction of nicotine to the brain is probably not sufficient to impact reward and indeed a recent study with NicVax, which had previously failed both phase 2 and phase 3 trials, showed vaccinated subjects had ~25% reduction of nicotine in their brains [9].

Antibody function depends on both the amount of antibody (titer) as well as the quality of the antibody (affinity), so further improvements in function could in theory be achieved with vaccines that induce even higher antibody titers and/or antibodies of greater affinity. Antibody titers require the antigen to be “immunogenic” but in our experience, are largely influenced by adjuvants, whereas affinity is largely determined by the antigen but can sometimes be influenced by adjuvants. Screening using functional assays is the best approach since the determinants of antibody responses are complex and the relative roles of titer and affinity are difficult to predict.

In order to render a small molecule such as nicotine immunogenic, it must be conjugated to a carrier protein, which provides a scaffold to present nicotine to the immune system, as well as the required T cell help to stimulate B cell proliferation and induce antibody class switching. A linker between the small molecule and the carrier as well as a ‘handle’ that can be used to improve the carrier protein functionality are also generally required. The identity of hapten [10–12], carrier protein [10,13], and linker [10,14] can all contribute to the immunogenicity of the conjugate antigen.

In this study, a series of novel nicotine-like haptens, were designed with variations in the attachment site on nicotine, the type of linker, and the handle used to attach the hapten to the carrier protein, which in all cases was DT. Mice were immunized with these hapten conjugates adjuvanted with CpG and aluminum hydroxide (Al(OH)_3_), and responses were compared by determination of Ab titers, affinity and function (using the radiolabeled nicotine challenge model). Based on these data, 5-amino-ethoxy-nicotine (Hapten 7) was selected and further used with the DT carrier to evaluate the influence of linker polarity, rigidity and length on immunogenicity and function in mice.

## Materials & Methods

### Ethics Statement

All animal studies were approved by the Institutional Animal Care and Use Committee of Pfizer Vaccine Research, Ottawa Laboratories in accordance with the guidelines of the Canadian Council on Animal Care (CCAC) and the Association for Assessment and Accreditation of Laboratory Animal Care (AAALAC).

### Antigens

#### Preparation of Haptens 1-11

**Figure 1:**
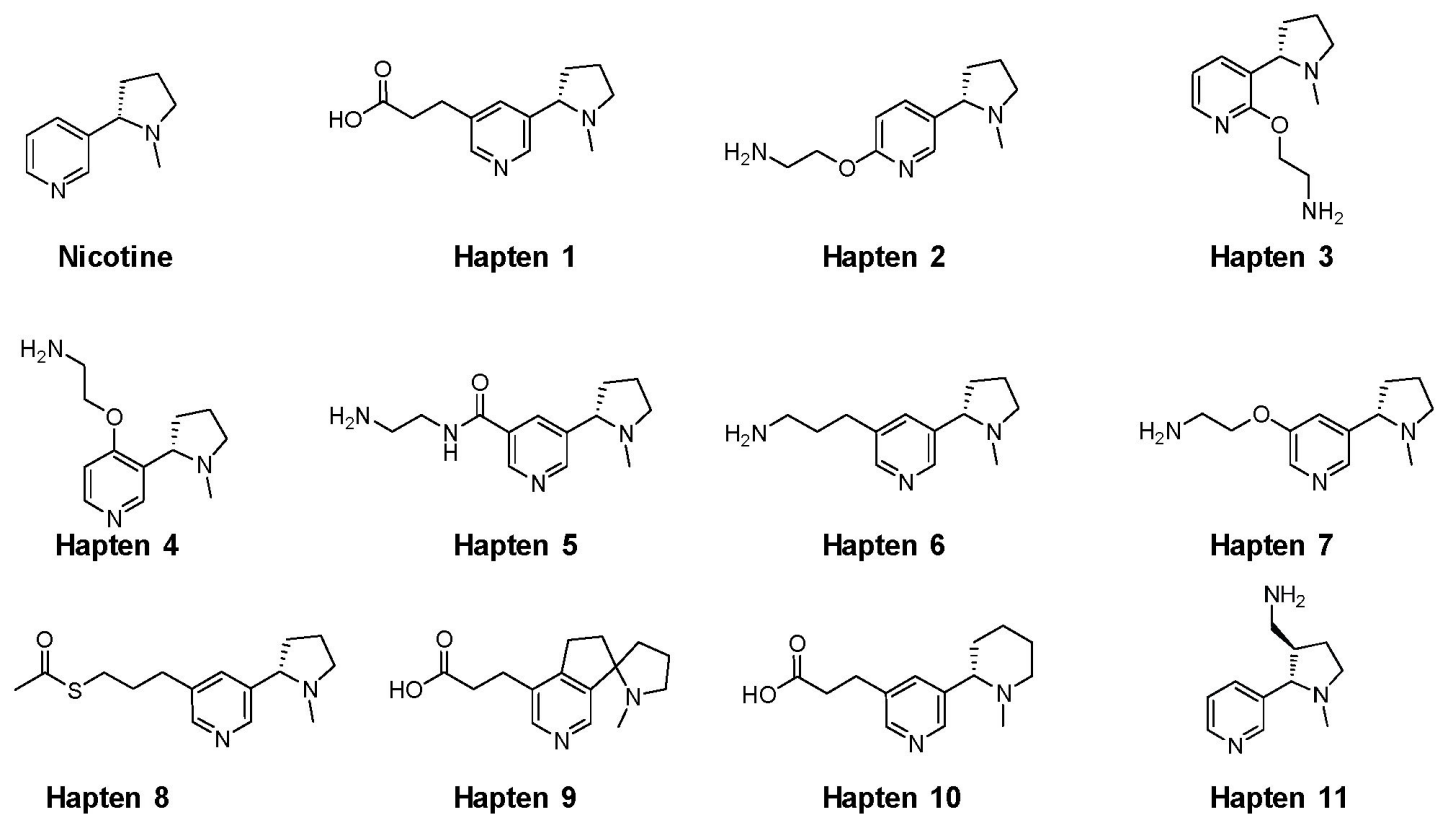
Nicotine and hapten structures. The thioacetate of Hapten 8 was deprotected to the thiol for coupling with DT.

A series of eleven nicotine-like haptens were tested which differed in the site of attachment of linker to nicotine, the nature of linker used, and the handle used to attach the hapten to the carrier protein (Figure 1). All compounds were prepared by Pfizer, with the exception of Hapten 11 (*trans*-3̕-aminomethylnicotine, 3̕AmNic) which was purchased from Toronto Research Chemicals, North York, Ontario, Canada. The routes to synthesize all other haptens are summarized below. Additional synthetic procedures for all compounds can be found in Supplementary Information.

**Figure 2:**
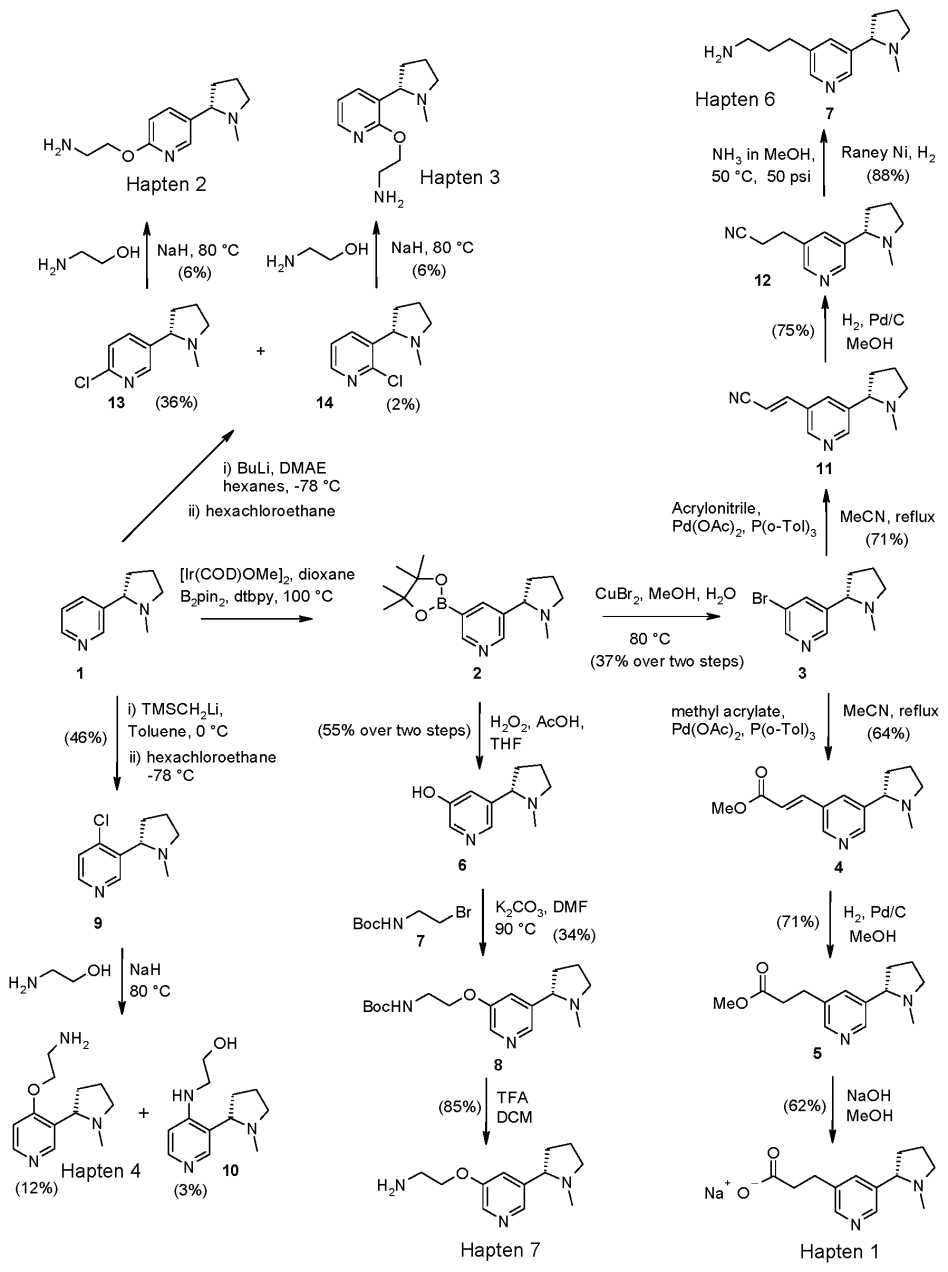
Synthesis of Haptens 1, 2, 3, 4, 6 and 7.

Haptens 1, 5, 6, 7 and 8 were all readily accessible via a common key intermediate, the boronate ester **2** (Figure 2); this intermediate could be readily synthesized as a single regioisomer from nicotine (1) utilizing the method of Hartwig [15] via iridium-mediated borylation. **2** was readily converted to bromide **3** using copper (II) bromide (CuBr_2_) (Figure S1). Alcohol **6** was accessed from **2** via oxidative cleavage with hydrogen peroxide (H_2_O_2_) and acetic acid (AcOH); in this reaction, the presence of the acid was key to prevent the pyridine nitrogen being oxidized (Figure S16, Figure S17). **6** was then alkylated with bromide **7** and deprotected to provide Hapten 7 (Figure S18, Figure S19). The bromide **3** was used to synthesize a range of linking groups which could be readily appended via palladium coupling. Heck coupling of methyl acrylate followed by hydrogenation and hydrolysis allowed access to the sodium salt of Hapten 1 (Figure S2, Figure S3, Figure S4) while coupling with acrylonitrile and reduction allowed access to Hapten 6 (Figure S5, Figure S6, Figure S7).

Functionalisation at the 2- and 6-positions of nicotine was achieved using the methodology of Comins [16]. Lithiation with *n*-butyl lithium /lithium dimethylethanolamine (*n*-BuLi/LiDMAE) [17], followed by quenching into hexachloroethane gave a mixture of 2- and 6-substituted chloronicotines **13** and **14** (Figure S11) which were converted to Haptens 2 and 3 respectively by heating with the sodium alkoxide of ethanolamine (Figure S12, Figure S13). Lithiation at the 4-position was accomplished using Trimethylsilylmethyllithium (TMSCH _2_Li) [18]; quenching with hexachloroethane gave chloride **9** (Figure S14). **S**ubsequent reaction with the sodium alkoxide of ethanolamine gave the 4-substituted Hapten 4 together with some undesired alcohol, **10** (Figure S15).

**Figure 3:**
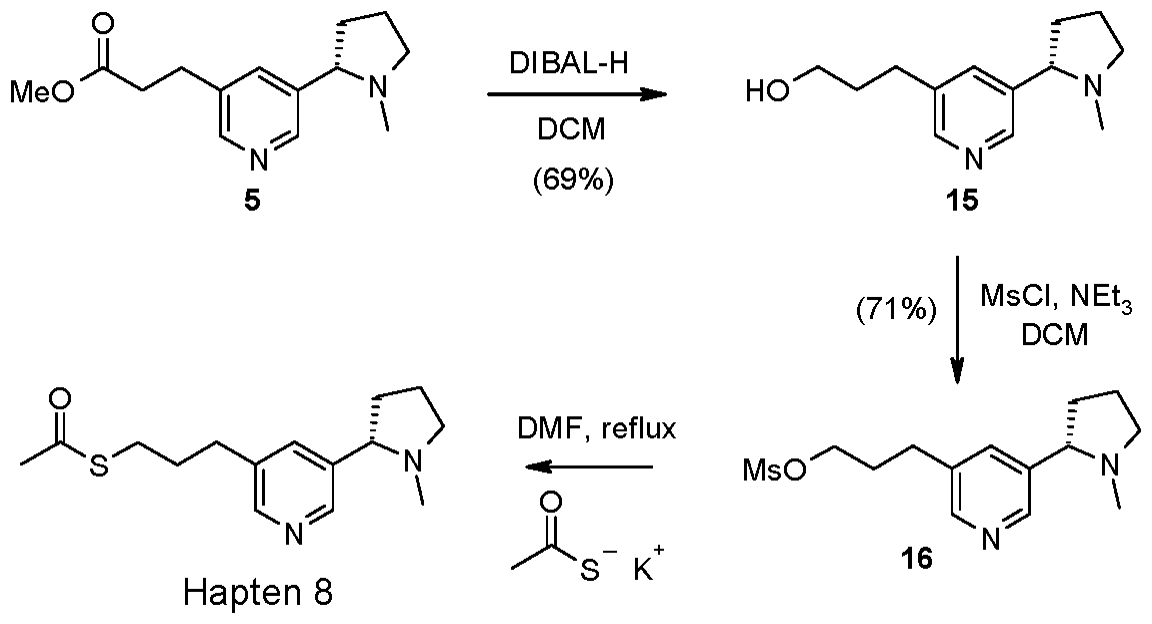
Synthesis of Hapten 8.

Ester **5** could be converted to the thioacetate Hapten 8 via reduction, mesylation and displacement with potassium thioacetate (Figure 3) (Figure S8, Figure S9, Figure S10). Cleavage of the acetate group was not carried out as the thiol was found to readily oxidise to the corresponding disulfide *in situ* and so the group was cleaved at the point of coupling to the carrier protein.

**Figure 4:**
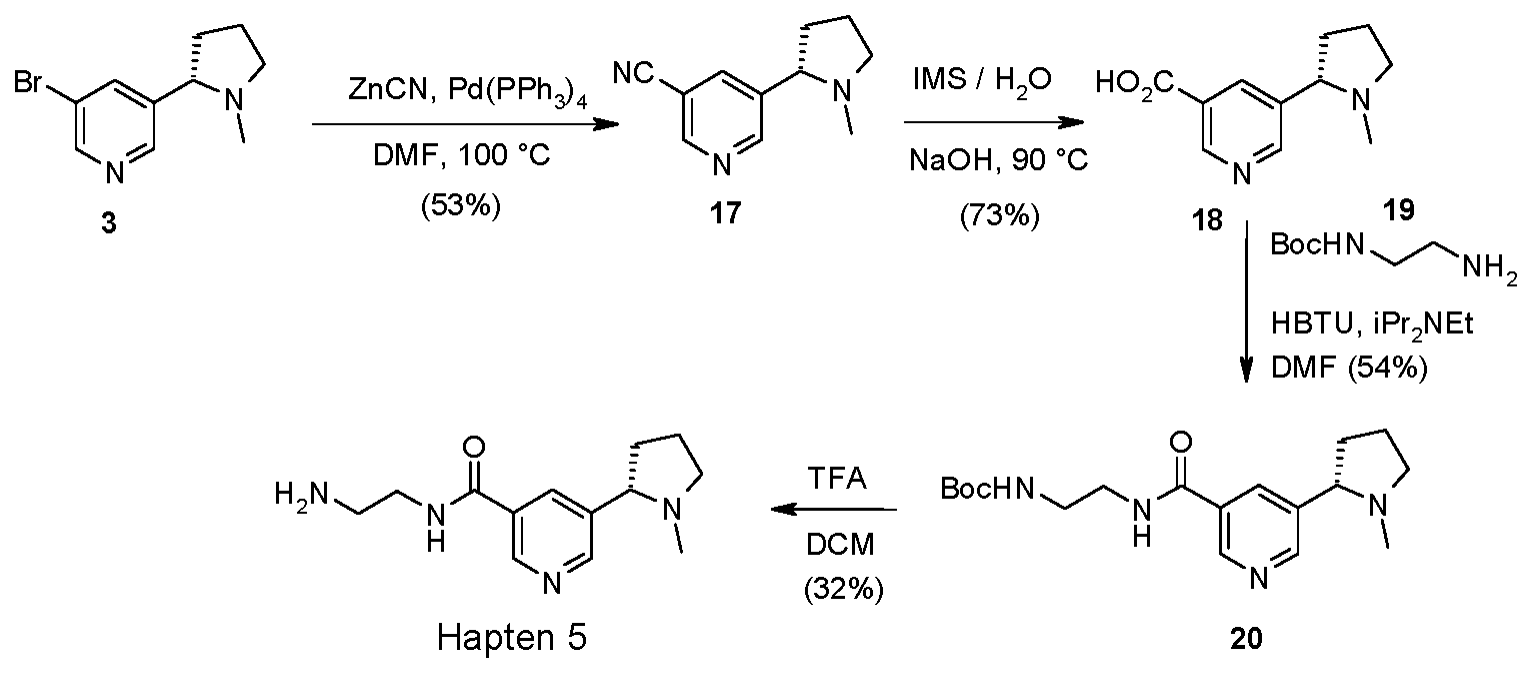
Synthesis of Hapten 5.

Palladium catalyzed coupling of zinc cyanide with bromide **3** allowed access to nitrile **17** which following hydrolysis, amide coupling and tert-butyloxycarbonyl (BOC) deprotection gave Hapten 5 (Figure 4) (Figure S24, Figure S25, Figure S26, Figure S27).

Hapten 9 was synthesized using a modification of the method of Ullrich [19].

**Figure 5:**
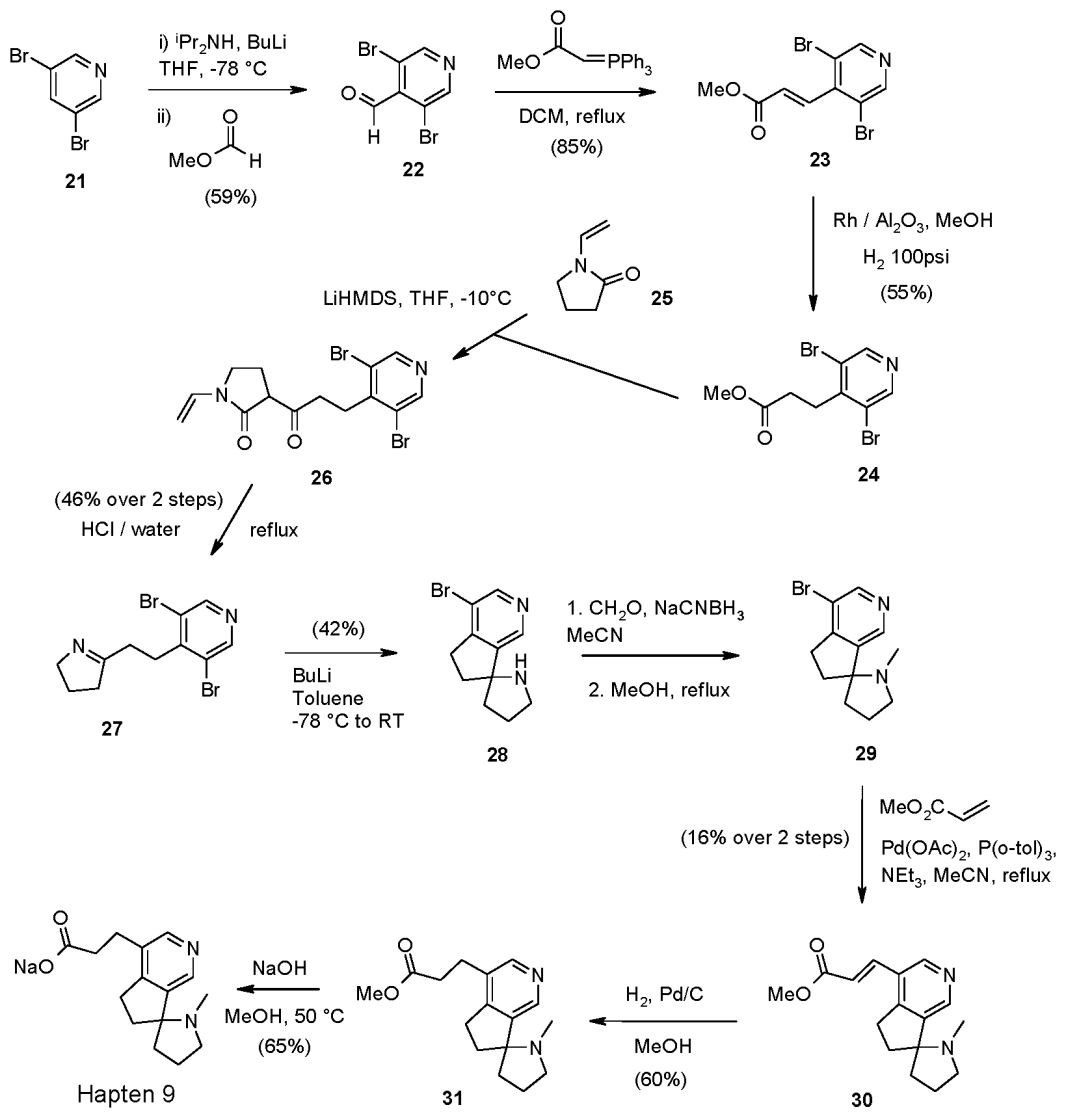
Synthesis of Hapten 9.

Dibromopyridine **21** was metallated with lithium diisopropylamide (LDA) and quenched with methyl formate to give aldehyde **22** (Figure S28) which was converted through to ester **24** via Wittig reaction and hydrogenation over rhodium on alumina (Figure 5) (Figure S29, Figure S30). Ester **24** was reacted with the enolate of lactam **25** to give ketoamide **26** (Figure S31). Acidic hydrolysis with concomitant decarboxylation and condensation gave imine **27** (Figure S32) which was metallated with butyllithium leading to *in situ* ring-closure to give racemic *spiro* amine **28** (Figure S33). Methylation under reductive amination conditions gave **29** (Figure S34) while subsequent Heck coupling, hydrogenation and basic hydrolysis generated Hapten 9 as a racemic mixture (Figure S35, Figure S36, Figure S37).

**Figure 6:**
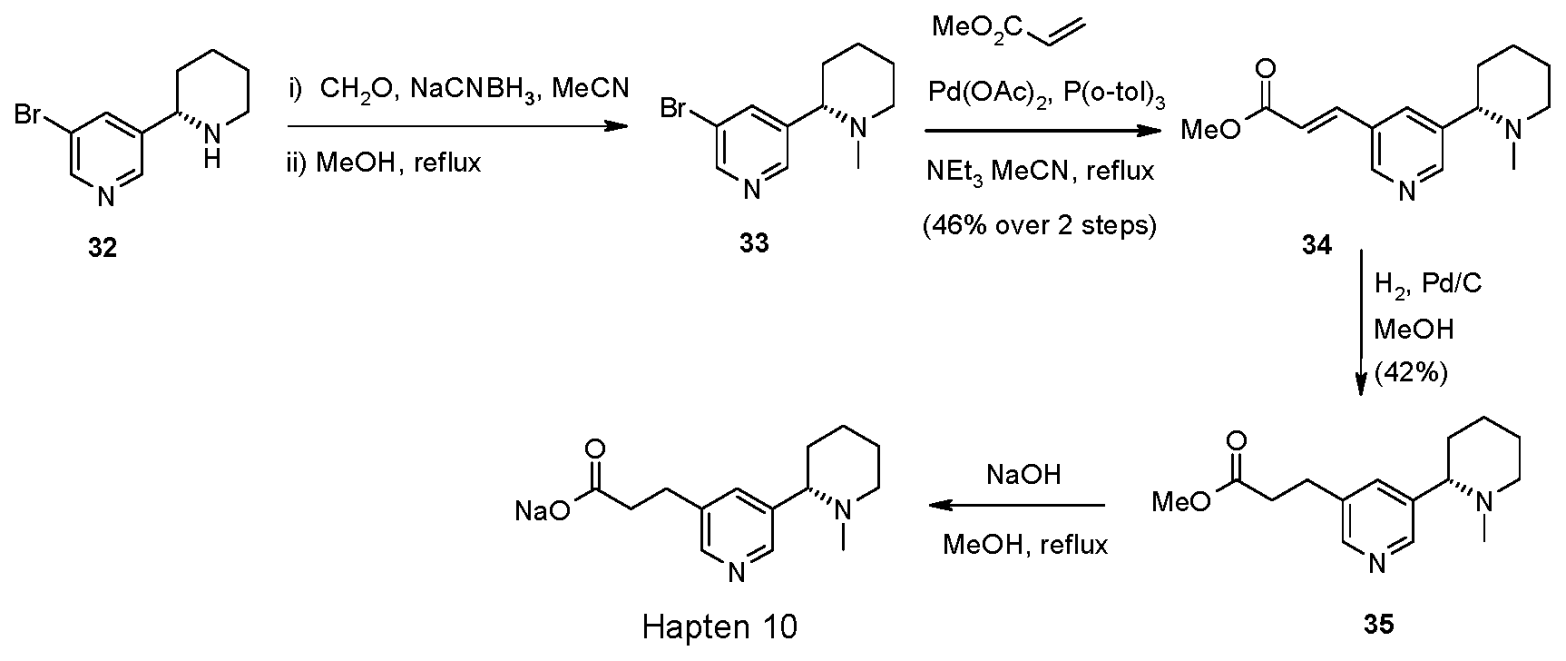
Synthesis of Hapten 10.

Hapten 10 was synthesised via the commercially available bromide **32** utilising similar methodology to the previous compounds (Figure 6) (Figure S20, Figure S21, Figure S22, Figure S23).

The degree of hapten coupling to DT was measured for all haptens using a reverse-phase HPLC (RP-HPLC) method utilizing a Waters C-18 X-Bridge column with a gradient of 0.1% triethylamine (TEA): 0.1% TEA in methanol (Table 1). In this process, haptens were uncoupled from DT by acid hydrolysis and analysis of pre-hydrolysis and post-hydrolysis levels used to determine the degree of conjugation per unit loading of DT.

**Table 1:** Hapten Loading of Different Hapten-DT Conjugates.

Hapten #	Hapten Loading (per unit DT)
1	5
2	14
3	19
4	3
5	15
6	19
7	13
8	N/A*
9	2
10	6
11	9

* N/A – not available

#### Preparation of Haptens 7.L1 to 7.L12: General Scheme

**Figure 7:**
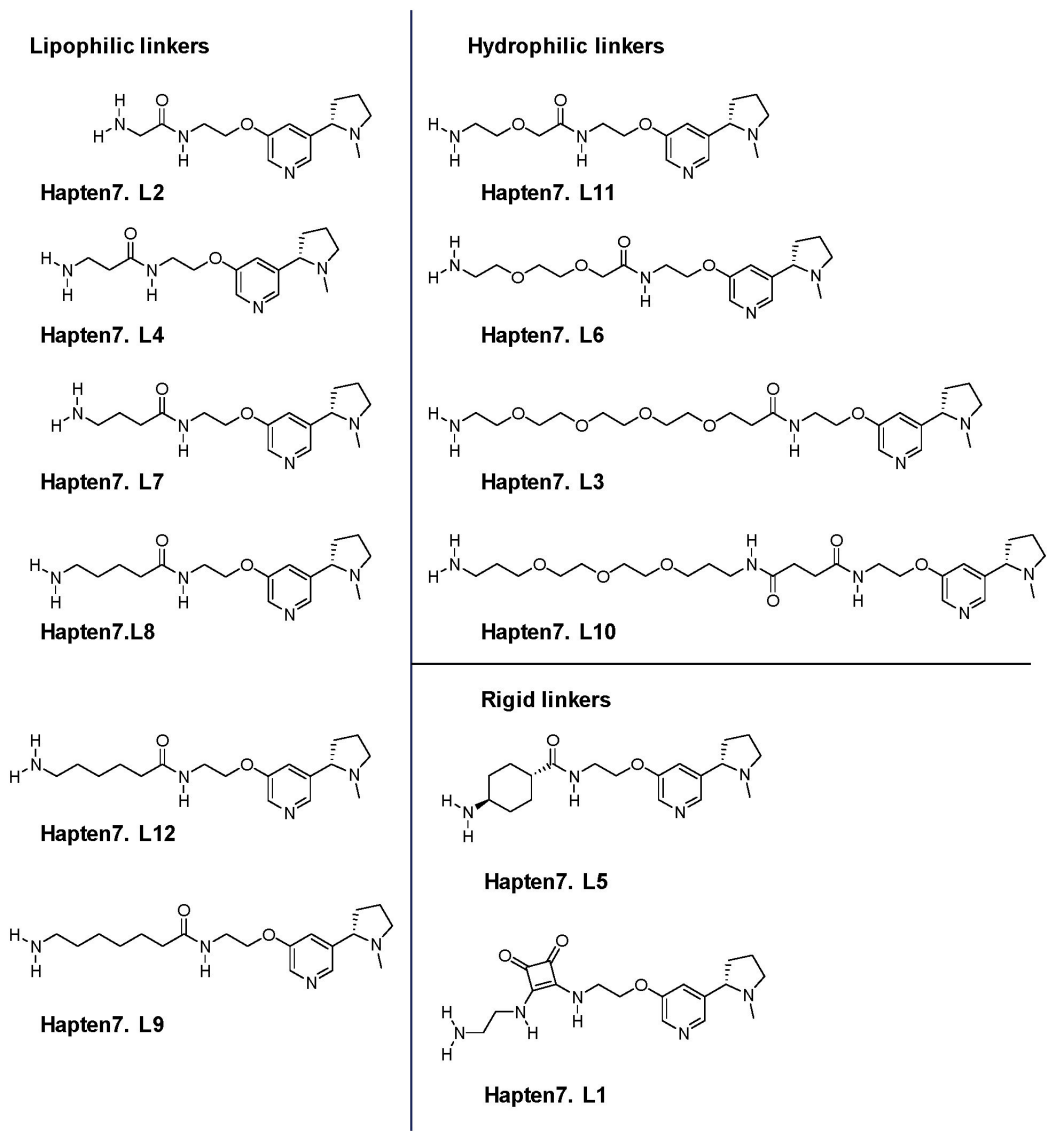
Structures of different linker-modified nicotine hapten.

**Figure 8:**
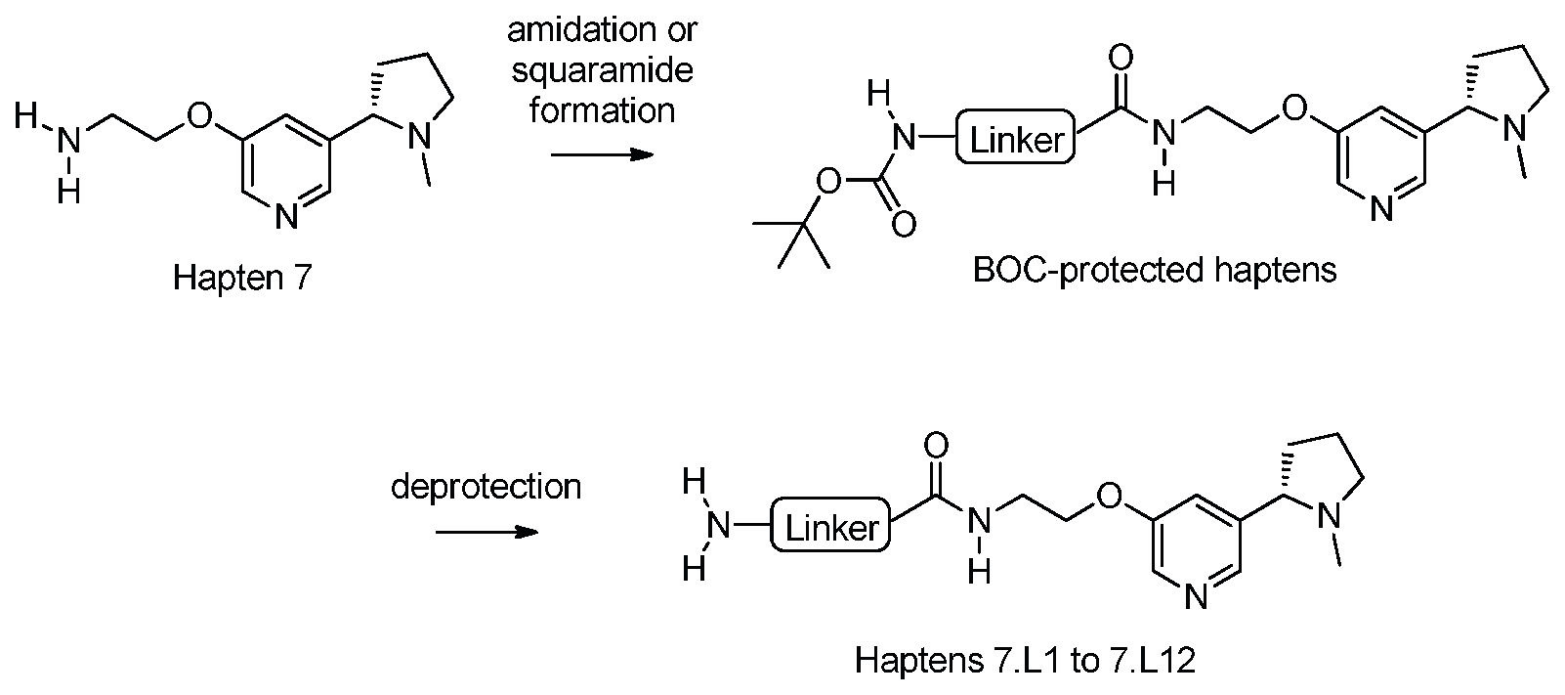
General scheme for addition of different linkers to Hapten 7.

Linkers of different polarity, length and rigidity were appended to Hapten 7 to yield a series of linker modified haptens (designated Hapten 7.L1 to Hapten 7.L12, Figure 7) using the general scheme depicted in Figure 8.

Haptens 7.L2 to 7.L12 were prepared according to the following general procedure:

Hapten 7 was dissolved in 2-methyltetrahydrofuran (2-MeTHF) to which was added the acid-functionalised linker, coupling agent 2,4,6-Tripropyl-1,3,5,2,4,6-trioxatriphosphorinane-2,4,6-trioxide (T3P^®^) and triethylamine (Et _3_N). The reactions were stirred at room temperature (RT) under nitrogen for 16 hours and were then evaporated *in vacuo* to leave orange oils. These were dissolved in methanol (MeOH) and run through a 5 g SCX cartridge eluting with MeOH, then 4:1 MeOH: Ammonia (7M in MeOH). The appropriate fractions were evaporated *in vacuo* and chromatographed on 4 g ISCO cartridges eluting with mixtures of dichloromethane (DCM), MeOH and ammonia (NH_3_) and evaporated *in vacuo* to yield the protected haptens that were used in the next step without further purification or characterization.

The protected version of Hapten 7.L1 was prepared from diethyl squarate, which was dissolved in ethanol (EtOH) and treated with BOC-ethylenediamine, Hünigs base and Hapten 7 and the reaction stirred at RT for 16 hours. The mixture was evaporated *in vacuo* to leave an orange oil that was dissolved in MeOH and run through a 5 g SCX cartridge eluting with MeOH, then 4:1 MeOH: Ammonia (7M in MeOH). The appropriate fractions were evaporated *in vacuo* and chromatographed as above.

BOC-protected amines were deprotected to yield Haptens 7.L1 to 7.L12 by dissolving in DCM and cooling to 0 °C. To this cooled solution, trifluoroacetic acid (TFA) was added and the reaction was allowed to warm to RT and stirred for 16 hours. The reaction mixtures were evaporated *in vacuo* to leave orange gums that were dissolved in MeOH and loaded onto 5 g SCX cartridges and eluted with MeOH, then 4:1 MeOH: Ammonia (7M in MeOH). The solutions were evaporated under reduced pressure to yield the haptens depicted in Figure 7.

The degree of hapten coupling to DT was measured for all haptens as outlined above (Table 2).

**Table 2:** Hapten Loading of Different Hapten 7-DT Conjugates.

Hapten #	Hapten Loading (per unit DT)
7.L1	19
7.L2	23
7.L3	21
7.L4	19
7.L5	4
7.L6	24
7.L7	20
7.L8	14
7.L9	12
7.L10	13
7.L11	29
7.L12	11

#### Conjugation of Haptens to Diphtheria Toxoid

Diphtheria Toxoid (DT; Pfizer, Lincoln, NE) in Dulbecco’s Phosphate Buffered Saline (DPBS, Gibco, Grand Island, NY) was first derivatised with succinic anhydride (Acros, Pittsburgh, PA) added as powder. The reaction mixture was incubated for two hours at RT and post-incubation excess succinic anhydride and reaction by-products were removed by buffer exchange into DPBS (using a Nap-25 desalting column, Pierce, Rockford, IL). An excess of the desired hapten (solubilised in DPBS) was added to the succinylated DT, along with an equal weight of 1-ethyl-3-(3-diethylamino)propyl carbodiimide hydrochloride (EDC) (Pierce, Rockford, IL) and sulfo *N*-hydroxysuccinimide (sNHS) (Sigma Aldrich, St Louis, MO). The conjugation reaction was incubated for two hours at room temperature to yield the hapten-DT conjugate. Post-conjugation, excess reagents and reaction bi-products were removed by buffer exchange into DPBS (Nap-25 Column, Pierce) prior to storage at 2-8 °C.

### Adjuvants

The adjuvants used were a B Class CpG ODN (CpG) of sequence 5’ TCG TCG TTT TTC GGT GCT TTT 3’, which was synthesized with a wholly nuclease-resistant phosphorothioate backbone (Pfizer, St. Louis, MI), and Al(OH)^3^ (Alhydrogel “85”, Brenntag Biosector, Denmark).

### Immunizations

Female BALB/c mice (Charles River Laboratories, Montreal, QC, n=10/group) were immunized with 10 µg of Hapten-DT conjugate in combination with Al(OH)_3_ (40 µg Al^3+^) and CpG (50 µg) made up to a total volume of 50 µl with phosphate buffered saline (PBS; Sigma Chemical Co., St. Louis, MO). Preliminary studies had demonstrated that these doses of antigen and adjuvants were appropriate for screening purposes. The vaccine formulations were administered by intramuscular (IM) injection in the left tibialis anterior (TA) muscle of mice lightly anaesthetized with Isoflurane® (CDMV, St. Hyacinthe, QC) on days 0, 28 and 42. Animals were bled on days 27, 41 and 54 by submandibular venus puncture using heparin as an anti-coagulant and recovered plasma used for quantitation of nicotine-specific immune responses. All groups were repeated on at least one occasion to ensure reproducibility of results.

Using a single Hapten-DT conjugate, female Sprague Dawley rats (Charles River Laboratories, Montreal, QC, n=5/group) were immunized with 0.4 mg of Hapten7-DT conjugate in combination with Al(OH)_3_ (0.4 mg Al^3+^) and CpG (0.5 mg) made up to a total volume of 500 µl with phosphate buffered saline (PBS; Sigma Chemical Co., St. Louis, MO). The vaccine formulations were administered as above on days 0, 28, 56, 84 and 112. Animals were bled two weeks following last dose and recovered plasma used for quantitation of nicotine-specific immune responses.

### Anti-nicotine antibody ELISA

The levels of anti-nicotine IgG antibodies in mouse plasma were quantified by endpoint ELISA (in triplicate) for individual animals using 96-well plates coated with a nicotine derivative (rac-trans-3’-thio methyl nicotine dihydrochloride) conjugated to bovine serum albumin as previously described [8]. Titers were calculated as the dilution corresponding to an absorbance reading 50% of the maximal value using GraphPad Prism (GraphPad Software, San Diego, CA).

### Antibody Affinity by Competition ELISA

The relative affinity of antibodies was determined by competition ELISA as previously described [8]. In brief, plasma samples, previously determined to contain anti-nicotine antibodies, were diluted to achieve absorbance values of approximately 1.0-1.5 at 450 nm; and nicotine was serially diluted starting at 20,000 µM. Equal volumes of diluted samples and nicotine were incubated for 1 hour at 37 °C, added to nicotine-BSA coated plates and antibody binding determined. OD readings at 450 nm were plotted against the molar concentration of nicotine and the 50% inhibition (IC_50_) was extrapolated for each sample tested. For rat samples, a modification of this assay was used whereby mean fluorescent intensity was measured using a Luminex-based microbead assay with nicotine added over a range of concentrations from 0.2 to 20 µM. Percent inhibition was calculated at each concentration of nicotine and the 50% inhibition (IC_50_) was interpolated for each sample tested.

### In Vivo Functional Assay: Nicotine Distribution in Brain and Plasma

The function of anti-nicotine Ab in immunized mice was evaluated by assessing nicotine distribution in the brain and plasma following tail vein infusion (<5 seconds) of 0.05 mg/kg of nicotine hydrogen tartrate (Sigma-Aldrich) containing 3 µCi ^3^H-nicotine (PerkinElmer) in 100 µL of PBS as previously described [8]. The 0.05 mg/kg dose of nicotine used is considered approximately equivalent to the mg/kg dose of nicotine delivered to a human by three smoked cigarettes [20].

### Ex Vivo Functional Assay: Nicotine-binding Capacity of Anti-nicotine Antibodies

The function of anti-nicotine Ab in immunized rats was evaluated by assessing nicotine-binding capacity using an equilibrium dialysis method [8]. In brief, plasma collected after vaccine dosing was spiked with a fixed amount of nicotine equivalent to high blood levels in smokers (100 ng/mL) and then subjected to equilibrium dialysis against DPBS for 3 h at 37°C using Spectrapor 4 membranes with a molecular weight cutoff of 12 to 14 kDa and 20 Micro-cell Equilibrium Dialyzer (Spectrum Labs, Rancho Dominguez, CA). The 100 ng/mL spiking dose of nicotine was selected as this represents a high concentration of nicotine in the arterial blood of a heavy smoker. Using nicotine-d3 as internal standard, aliquots from the sample and buffer sides of dialysis membrane were extracted using a protein precipitation extraction procedure. The extracted samples were then injected into an HPLC equipped with Applied Biosystems API 4000 LC/MS/MS system and concentrations of bound and unbound nicotine determined.

### Statistical analysis

Data were analyzed using GraphPad Prism. Statistical significance of the difference between groups was calculated by 1-factor analysis of variance (ANOVA) followed by post-hoc analysis. Differences were considered to be not significant with p>0.05.

## Results

### Effect of Hapten Design on Immunogenicity of Hapten-DT conjugates

Pilot studies showed very poor antibody responses were induced in mice when conjugates were administered without an adjuvant, therefore for comparison of conjugates in this study, each was administered together with aluminum hydroxide and CpG adjuvants.

**Figure 9:**
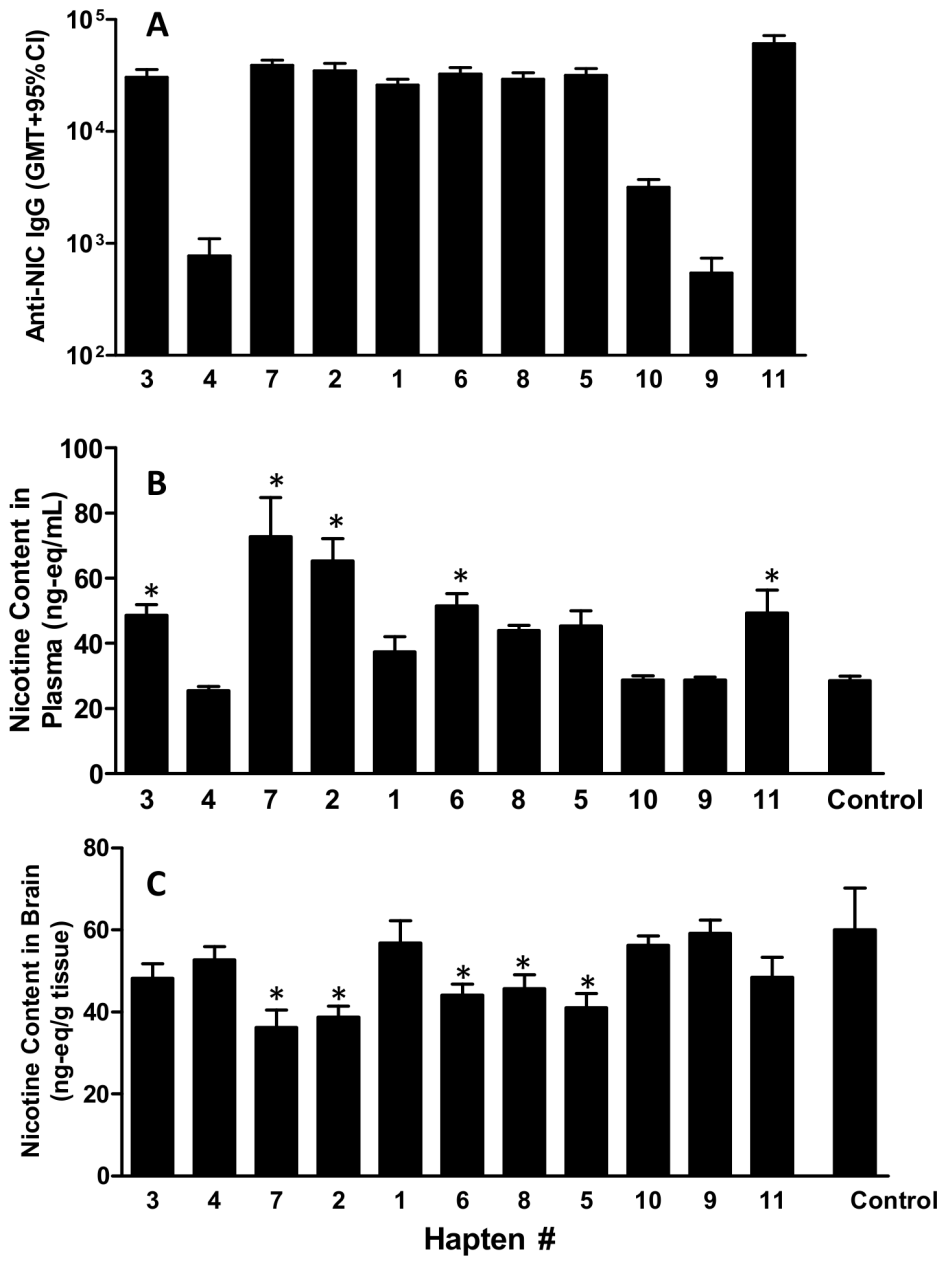
Anti-nicotine antibody titer and function in mice. Panel A: BALB/c mice (n = 12/gp) were immunized by IM injection with 10 µg of different nicotine Hapten-DT conjugates adjuvanted with Al(OH)_3_ (40 µg Al^3+^) + CpG 24555 (50 µg) on days 0, 28 and 42. Plasma was collected on day 54 and anti-nicotine antibody levels determined by ELISA (Panel A). On day 56 animals received an IV injection of ^3^H-nicotine (0.05 mg/kg) and plasma and brains collected. Panel B shows nicotine levels in plasma (ng-eq/mL), and Panel C shows nicotine levels in brain (ng-eq/g).

All adjuvanted nicotine hapten conjugates generated nicotine-specific antibodies in mice, however levels varied depending on hapten design (p = 0.0002) (Figure 9, Panel A). Overall, the highest levels of nicotine-specific IgG were obtained with Hapten 11-DT (p<0.05 compared to all other conjugates), although Haptens 1, 2, 3, 5, 6, 7 or 8 DT conjugates were also able to induce high levels of anti-nicotine antibodies (titer ≥10^5^). Lowest levels were induced by Hapten 4-DT and Hapten 9-DT (p<0.05 compared to all other conjugates). In addition, antibody affinity varied depending on hapten conjugate tested with equivalent affinity (<20 µM) induced by conjugates containing Haptens 1, 2, 3, 5, 6, 7, 8 and the weakest affinity antibodies (>150 µM) induced by those containing Haptens 10 and 11 (Table 3). Ab titers for conjugates containing Haptens 4 or 9 were too low to determine affinity.

**Table 3:** Relative Affinity of Anti-Nicotine Antibodies induced by Different Hapten-DT Conjugates in Mice.

Hapten #	IC_50_ (µM)	SEM
1	19.2	3.2
2	8.7	0.7
3	15.4	1.6
5	15.2	3.6
6	18.7	1.8
7	20.9	8.0
8	17.4	5.9
10	163.4	11.6
11	211.2	86.8

The *in vivo* function of the anti-nicotine antibodies in mice, as assessed by IV administration of radiolabeled nicotine, also varied with different hapten conjugates (p < 0.0001 and p = 0.0002, for plasma and brain, respectively). Hapten conjugates containing Haptens 2, 3, 6, 7 and 11 significantly increased levels of nicotine in the blood compared to non-immunized animals (p<0.05) (Figure 9B), and hapten conjugates containing Haptens 2, 5, 6, 7 and 8 significantly decreased levels of nicotine in the brain (p<0.05) (Figure 9C). Overall best responses were obtained with conjugates containing Haptens 2, 6 or 7 that had ~130, 81 and 156% greater retention of nicotine in the plasma, and 36, 27 and 40% decrease of nicotine in the brain, respectively, as compared to control animals.

As expected, Ab function depended on both Ab titer and affinity with the best responses being obtained with conjugates that induced both high Ab levels and good affinity. In contrast, conjugates that induced either low Ab levels or poor affinity, even in the presence of high titers (e.g., Hapten 11-DT) resulted in poor functional results. Not every conjugate that induced good Ab responses and affinity resulted in good function, and it is possible this resulted from the relatively low sensitivity of the ELISA assay used to measure affinity in this study; in more recent studies we have found improved differentiation of affinity with Luminex based methods.

Hapten loading was determined for all conjugates (Table 1) and compared with antibody titer, affinity and function. However, while there was some degree of correlation with Ab function, as measured by concentration of nicotine in the brain (r^2^ = 0.55), there was only a weak correlation with antibody levels (r^2^ = 0.34) and no correlation with antibody affinity (r^2^ = 0.04).

### Effect of Linker on Immunogenicity of Hapten 7-DT conjugates

**Figure 10:**
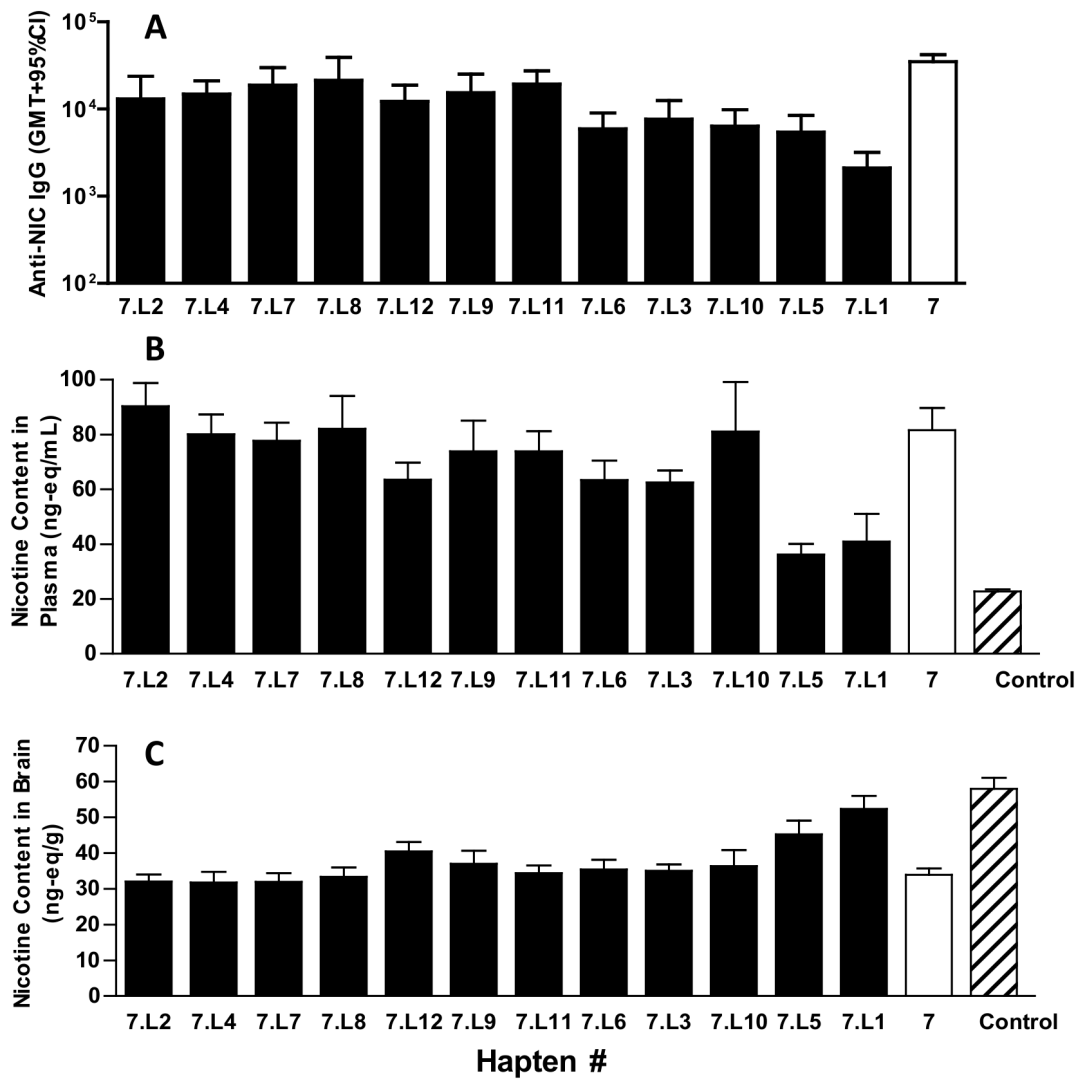
Anti-nicotine antibody titer and function in mice. Panel A: BALB/c mice (n = 12/gp) were immunized by IM injection with 10 µg of different nicotine Hapten-DT conjugates adjuvanted with Al(OH)_3_ (40 µg Al^3+^) + CpG 24555 (50 µg) on days 0, 28 and 42. Plasma was collected on day 54 and anti-nicotine antibody levels determined by ELISA (Panel A). On day 56 animals received an IV injection of ^3^H-nicotine (0.05 mg/kg) and plasma and brains collected. Panel B shows nicotine levels in plasma (ng-eq/mL), and Panel C shows nicotine levels in brain (ng-eq/g).

Since strong functional results were obtained with the Hapten 7-DT conjugate, Hapten 7 was chosen to investigate the effect of additional linker modifications including effect of polarity, length and rigidity. All linker modified Hapten 7 conjugates generated nicotine-specific Abs in mice, but levels varied depending on linker used (p < 0.0001) (Figure 10). None of the linker modified Hapten 7 conjugates gave levels of nicotine-specific Abs that were higher than those obtained with unmodified Hapten 7-DT, and those conjugates having rigid linkers (Hapten 7.L5, Hapten7.L1), or longer chain hydrophilic linkers (Hapten 7.L3, Hapten 7.L6 and Hapten 7.L10) gave significantly lower Ab levels (p<0.05). There was no significant difference in affinity between the linker modified Hapten 7 conjugates and Hapten 7-DT (p>0.05), with all having a measured IC_50_ of ~20 µM.

The *in vivo* function of the anti-nicotine Abs also varied with different linker modified Hapten 7 conjugates (p < 0.0001, for plasma and brain) (Figure 10). Although most of the linker modified Hapten 7 conjugates (with the exception of Hapten 7-L1 and Hapten 7.L5) resulted in decreased nicotine in the brain and increased nicotine in the plasma compared to control animals (p<0.05), none of them gave a better functional response than was obtained with Hapten 7-DT that had ~220% greater retention of nicotine in the plasma, and 60% decrease of nicotine in the brain, respectively, as compared to control animals. Hapten 7 conjugates with rigid linkers (Hapten 7.L5, Hapten 7.L1), gave a significantly weaker response than was obtained with Hapten 7-DT (p<0.05).

Hapten loading was determined for all conjugates (Table 2) and compared with antibody titer and function. However, there was no correlation observed with either antibody levels (r^2^ = 0.02) or antibody function, as measured by concentration of nicotine in the brain (r^2^ = 0.14), most likely since most conjugates had a similar levels of hapten loading.

Since best responses were obtained in mice using the Hapten 7-DT conjugate without the use of an additional linker, this conjugate was also evaluated in rats to assess the nicotine-binding capacity using an equilibrium dialysis method. At a spiking dose of 100 ng/mL (equivalent to high blood levels in smokers), approximately 100% of nicotine (95 ± 3.5%) was bound in serum from immunized rats with a mean IC_50_ of 0.95 ± 1.31 µM.

## Discussion

In recent years a number of anti-nicotine vaccines have been evaluated in the clinic. Of these, the greatest experience has been with NicVax (Nabi Pharmaceuticals) followed by NicQb (Cytos Biotechnology). Despite failure to improve long-term quit rates in the intent-to-treat population, there was some evidence to support the putative mechanism in that a subset of subjects with the highest Ab titers had significantly higher quit rates at 1 year compared to placebo [7]. It appears that both vaccines were developed to induce high anti-nicotine Ab titers so it is possible that, assuming the putative mechanism is sound, low affinity Ab may have contributed to the poor efficacy. It should also be noted that with both NicVax and NicQb, as is the case in all anti-nicotine vaccines, antibodies are generated against nicotine-derived haptens and not against nicotine per se. Thus, a high antibody titer generated by such vaccines may not necessarily reflect a strong ability to bind to nicotine in the blood; this highlights the importance of screening using functional assays such as drug distribution following nicotine challenge rather than just immunogenicity read-outs.

In attempts to improve anti-nicotine vaccine efficacy, a number of different approaches have been used including modifications of hapten structure [10–12], composition and position of linker [10,14], carrier protein [10,13], adjuvant [21], route of administration [21,22] and co-administration of more than one nicotine hapten conjugate [13]. All of these approaches have to some extent altered the immunogenicity of nicotine vaccines, at least in pre-clinical models. We have previously shown that adjuvant combinations such as Al(OH)_3_ and CpG can enhance the function of anti-nicotine vaccines through augmenting both antibody titer and affinity [8]. Herein using the same adjuvant combination previously tested (CpG/Al(OH)_3_), we evaluated the impact of antigen design on functional responses.

The pyrrolidine ring of nicotine has most commonly been used as the site of linker attachment to nicotine. The 3̕-position was first used to generate trans-3̕-succinyl-methyl nicotine by Langone et al in the early 1970s in attempts to create radioimmunoassay detection reagents [23], and since then the pyrrolidine ring has been used as the site of linker attachment in many pre-clinical anti-nicotine vaccine studies [11,22,24–27]. . Both NicVax and NicQb, utilize conjugation via the 3̕-position on the pyrrolidine ring of nicotine to *Pseudimonas aeroginosa* recombinant exoprotein A (NicVax) and a virus-like particle of bacteriophage Qb (NicQb), respectively. In our study, we evaluated the same nicotine hapten as used in NicVax (3̕AmNic) conjugated to DT via the 3̕-position on the pyrrolidine ring (Hapten 11), and compared this to a number of different haptens which varied in hapten design, linker, handle and site of attachment but which were all attached to DT via the pyridine ring. Of the 11 haptens tested, Hapten 11 induced the highest levels of Ab, but this did not translate to best functional responses. Previous studies have suggested that higher antibody titers can compensate for weaker affinity [12], however our data showed that even very high titers of low affinity antibodies were functionally inferior to lower titers of higher affinity antibodies. Our results suggested that an intact and unhindered pyrrolidine ring was essential for good functional responses since conjugates whose haptens had the pyrrolidine ring replaced by piperidine (Hapten 10), constrained (Hapten 9) or used as site of attachment to DT (Hapten 11) resulted in poor immunogenicity (low Ab titer and affinity) and function (high nicotine in brain). Poor responses were also obtained with Hapten 4, which was attached to DT at the 4-position of the pyridine ring, the closest available position to the pyrrolidine ring. In contrast, Hapten 3 which was linked to DT through the 2-position, distal to that used for Hapten 4, had a very different Ab and affinity response. Previous studies have shown that constrained haptens can increase both antibody titer and affinity compared to unconstrained haptens, however our data do not support this [12]. Differences in how the hapten is constrained as well as carrier and adjuvants may account for this discrepancy.

It was previously proposed that attachment to nicotine via the pyridine ring was preferable as this might increase the specificity for antibodies for nicotine rather than its metabolites [28]. Haptens 2, 3, 4 and 7 were all conjugated via the pyridine ring using the same aminoethoxy linker at each of the four different free positions on the pyridine ring (positions 6, 2, 4 and 5 respectively), and when tested in mice, the best response overall was obtained with linker attachment at the 5-position, good responses at the 6- or 2-positions and poor responses at the 4-position. These results indicate that not only is the ring important but also the exact site of linkage on that ring. Similar results have been reported previously, although in that case the 6-position was deemed best in contrast to the 5-position in the present study [14]. This discrepancy may be partly due to the different linkers and readouts used in the two studies.

The impact of different linkers and chemical handles on the 5-position as site of attachment was evaluated by comparing aminoethoxy (Hapten 7), aminopropyl (Hapten 6), carboxyl ethyl (Hapten 1), propanethiol (Hapten 8), and an amide-containing linker (Hapten 5). The best functional response was obtained with the aminoethoxy linker, although good responses were also obtained using the aminopropyl linker, whereas weaker responses were seen with other linkers/handles.

Hapten loading has previously been shown to be influential in determining immunogenicity of conjugate vaccines [29]. We demonstrated that hapten loading correlated to some extent with vaccine immunogenicity, suggesting that responses with some of our poorer conjugates could have been improved had hapten loading been optimized.

Linker length and flexibility have previously been reported as influential in determining the efficacy of anti-nicotine [10,14] and anti-methamphetamine vaccines [30], and in the elicitation of functional monoclonal Abs that bind methamphetamine [31,32]. Therefore we systematically investigated a series of different linkers which varied in length, polarity and flexibility. The synthetic complexity of the linker-hapten construct was also taken into consideration during the vaccine design, such that difficult-to-synthesize longer linkers were not pursued. Interestingly, we saw no significant improvement in responses with the additional linkers, and, in particular with the use of hydrophilic linkers, increased length appeared to result in the induction of weaker immune responses, possibly in an analogous manner to the reduced immunogenicity of PEGylated proteins [33]. Our weakest responses were obtained with the use of rigid linkers, namely the cyclohexyl (Hapten 7.L5) and squaramide tethers (Hapten 7.L1) suggesting that Ab generation and avidity is favoured by shorter and more flexible linkers. Previous studies using a derivative of Lipid A conjugated to KLH incorporating a similar bis-cyclohexyl linker to that used in Hapten7. L5 have demonstrated that active immunization with conjugates containing this linker could enhance antibody levels and induce significant protection against the effects of *E. Coli* lipid A in a murine model [34]. Also, squaramide-based coupling chemistry has been used extensively in the area of bioconjugation [35] and a squaramide-containing reactive immunogen has been used previously to generate monoclonal antibodies as tools for the investigation of paraoxon poisoning [36]. It is possible that the use of these linkers may have shifted the dominant epitope away from the nicotine hapten and onto the linker itself (especially as the squaramide motif is formally aromatic in nature); however, confirmation of this hypothesis was outside the scope of the current study. In the current study, a single carrier protein was used to evaluate multiple haptens and linkers. However, carrier itself can influence the immunogenicity of the conjugate and it is possible that the relative effects of the different linkers may have differed with a different carrier. In this study in rats, using Hapten7 conjugated to DT, we were able to induce antibodies with 100% nicotine binding at 100 ng/mL. However, in separate studies in non-human primates, using a modified Hapten7 conjugate vaccine with the same adjuvant combination, we have subsequently been able to obtain 100% binding with a ten-fold higher amount of nicotine (1000 ng/mL) [37].

Overall, these results demonstrate that immune responses induced in mice by nicotine-conjugate antigens are greatly influenced by hapten design including site of attachment of linker to nicotine, the nature of linker used, and the handle used to attach the hapten to DT. While both Ab titer and affinity contributed to function, affinity was more sensitive to antigen differences. Our work suggests that, assuming the basic concept of anti-nicotine vaccines is sound, better clinical outcomes may be realized using antigens selected for their ability to induce antibodies of both high titer and high affinity, especially when combined with adjuvants that strongly enhance both aspects.

## Supporting Information

Figure S1 **Preparation of (*S*)-3-bromo-5-(1-methylpyrrolidin-2-yl)pyridine.**
(TIF)

Figure S2 **Preparation of (*S*,*E*)-methyl 3-(5-(1-methylpyrrolidin-2-yl)pyridin-3-yl)acrylate.**
(TIF)

Figure S3 **Preparation of (S)-methyl 3-(5-(1-methylpyrrolidin-2-yl)pyridin-3-yl)propanoate.**
(TIF)

Figure S4 **Preparation of (*S*)-3-(5-(1-methylpyrrolidin-2-yl)pyridin-3-yl)propanoic acid (Hapten 1).**
(TIF)

Figure S5 **Preparation of (*S*)-3-(5-(1-methylpyrrolidin-2-yl)pyridin-3-yl)acrylonitrile.**
(TIF)

Figure S6 **Preparation of (*S*)-3-(5-(1-methylpyrrolidin-2-yl)pyridin-3-yl)propanenitrile.**
(TIF)

Figure S7 **Preparation of (*S*)-3-(5-(1-methylpyrrolidin-2-yl)pyridin-3-yl)propan-1-amine (Hapten 6).**
(TIF)

Figure S8 **Preparation of (*S*)-3-(5-(1-methylpyrrolidin-2-yl)pyridin-3-yl)propan-1-ol.**
(TIF)

Figure S9 **Preparation of (*S*)-3-(5-(1-methylpyrrolidin-2-yl)pyridin-3-yl)propyl methanesulfonate.**
(TIF)

Figure S10 **Preparation of (S)-S-3-(5-(1-methylpyrrolidin-2-yl)pyridin-3-yl)propyl ethanethioate (Hapten 8).**
(TIF)

Figure S11 **Preparation of (*S*)-2-chloro-5-(1-methylpyrrolidin-2-yl)pyridine or 6-chloronictone and (*S*)-2-chloro-3-(1-methylpyrrolidin-2-yl)pyridine or 2-chloronicotine.**
(TIF)

Figure S12 **Preparation of (*S*)-2-(5-(1-methylpyrrolidin-2-yl)pyridin-2-yloxy)ethanamine (Hapten 2).**
(TIF)

Figure S13 **Preparation of (*S*)-2-(3-(1-methylpyrrolidin-2-yl)pyridin-2-yloxy)ethanamine (Hapten 3).**
(TIF)

Figure S14 **Preparation of (*S*)-4-chloro-3-(1-methylpyrrolidin-2-yl)pyridine or 4-chloronicotine.**
(TIF)

Figure S15 **Preparation of (*S*)-2-(3-(1-methylpyrrolidin-2-yl)pyridin-4-yloxy)ethanamine (Hapten 4) and (*S*)-2-(3-(1-methylpyrrolidin-2-yl)pyridin-4-ylamino)ethanol.**
(TIF)

Figure S16 **Preparation of (*S*)-3-(4-methoxybenzyloxy)-5-(1-methylpyrrolidin-2-yl)pyridine.**
(TIF)

Figure S17 **Preparation of (*S*)-5-(1-methylpyrrolidin-2-yl)pyridin-3-ol or 5-hydroxynicotine.**
(TIF)

Figure S18 **Preparation of (S)-tert-butyl 2-(5-(1-methylpyrrolidin-2-yl)pyridin-3-yloxy)ethylcarbamate.**
(TIF)

Figure S19 **Preparation of (*S*)-2-(5-(1-methylpyrrolidin-2-yl)pyridin-3-yloxy)ethanamine (Hapten 7).**
(TIF)

Figure S20 **Preparation of (*S*)-3-bromo-5-(1-methylpiperidin-2-yl)pyridine.**
(TIF)

Figure S21 **Preparation of (S,*E*)-methyl 3-(5-(1-methylpiperidin-2-yl)pyridin-3-yl)acrylate.**
(TIF)

Figure S22 **Preparation of (S)-methyl 3-(5-(1-methylpiperidin-2-yl)pyridin-3-yl)propanoate.**
(TIF)

Figure S23 **Preparation of (*S*)-3-(5-(1-methylpiperidin-2-yl)pyridin-3-yl)propanoic acid, sodium salt (Hapten 10).**
(TIF)

Figure S24 **Preparation of (*S*)-5-(1-methylpyrrolidin-2-yl)nicotinonitrile or 5-cyanonicotine.**
(TIF)

Figure S25 **Preparation of (*S*)-5-(1-methylpyrrolidin-2-yl)nicotinic acid.**
(TIF)

Figure S26 **Preparation of (S)-tert*-*butyl 2-(5-(1-methylpyrrolidin-2-yl)nicotinamido)ethylcarbamate.**
(TIF)

Figure S27 **Preparation of (S)-N-(2-aminoethyl)-5-(1-methylpyrrolidin-2-yl)nicotinamide (Hapten 5).**
(TIF)

Figure S28 **Preparation of 3,5-dibromoisonicotinaldehyde.**
(TIF)

Figure S29 **Preparation of (E)-methyl 3-(3,5-dibromopyridin-4-yl)acrylate.**
(TIF)

Figure S30 **Preparation of Methyl 3-(3,5-dibromopyridin-4-yl)propanoate.**
(TIF)

Figure S31 **Preparation of 3-(3-(3,5-dibromopyridin-4-yl)propanoyl)-1-vinylpyrrolidin-2-one.**
(TIF)

Figure S32 **Preparation of 3,5-dibromo-4-(2-(3,4-dihydro-2H-pyrrol-5-yl)ethyl)pyridine.**
(TIF)

Figure S33 **Preparation of 4-bromo-5,6-dihydrospiro[cyclopenta[c]pyridine-7,2'-pyrrolidine].**
(TIF)

Figure S34 **Preparation of 4-bromo-1'-methyl-5,6-dihydrospiro[cyclopenta[c]pyridine-7,2'-pyrrolidine].**
(TIF)

Figure S35 **Preparation of (E)-methyl 3-(1'-methyl-5,6-dihydrospiro[cyclopenta[c]pyridine-7,2'-pyrrolidine]-4-yl)acrylate.**
(TIF)

Figure S36 **Preparation of Methyl 3-(1'-methyl-5,6-dihydrospiro[cyclopenta[c]pyridine-7,2'-pyrrolidine]-4-yl)propanoate.**
(TIF)

Figure S37 **Preparation of 3-(1'-methyl-5,6-dihydrospiro[cyclopenta[c]pyridine-7,2'-pyrrolidine]-4-yl)propanoic acid sodium salt (Hapten 9).**
(TIF)
